# Impact of Multi-Micronutrient Supplementation on Growth and Morbidity of HIV-Infected South African Children

**DOI:** 10.3390/nu5104079

**Published:** 2013-10-11

**Authors:** Siyazi Mda, Joop M. A. van Raaij, François P. R. de Villiers, Frans J. Kok

**Affiliations:** 1 Division of Human Nutrition, Wageningen University and Research Centre, P.O. Box 8129, Wageningen 6700EV, The Netherlands; E-Mails: joop.vanraaij@wur.nl (J.M.A.R.); frans.kok@wur.nl (F.J.K.); 2 Department of Paediatrics and Child Health, University of Limpopo, Medunsa Campus, P.O. Box 221, Medunsa 0204, South Africa; E-Mail: alfafrancois@yahoo.co.uk

**Keywords:** multi-micronutrients, HIV, growth, diarrhea, respiratory infections, children

## Abstract

Poor growth, micronutrient deficiencies and episodes of diarrhea and respiratory infections occur frequently in HIV-infected children. We investigated whether multi-micronutrient supplementation would improve the growth performance and reduce the number of episodes of diarrhea and/or of respiratory symptoms in HIV-infected children. In a double-blind randomized trial, HIV-infected South African children aged 4–24 months (*n* = 201) were assigned to receive multi-micronutrient supplements or placebo daily for six months. The children were assessed for respiratory symptoms or diarrhea bi-weekly; weights and heights were measured monthly. In total, 121 children completed the six month follow up study period (60%). A total of 43 children died; 27 of them had received supplements. This difference in mortality was not statistically significant (*p* = 0.12). Weight-for-height Z-scores improved significantly (*p* < 0.05) among children given supplements compared with those given placebo (0.40 (0.09–0.71)) *versus* −0.04 (−0.39–0.31) (mean (95% CI)). Height-for-age *Z*-scores did not improve in both treatment groups. The number of monthly episodes of diarrhea in the placebo group (0.36 (0.26–0.46)) was higher (*p* = 0.09) than in the supplement group (0.25 (0.17–0.33)) and the number of monthly episodes of respiratory symptoms was significantly higher (*p* < 0.05) among children on placebos (1.01 (0.83–1.79)) than those on supplements (0.66 (0.52–0.80)). Multi-micronutrient supplements significantly improved wasting and reduced the number of episodes of diarrhea and respiratory symptoms.

## 1. Introduction

In 2011, there were 330,000 children younger than 15 years worldwide who were newly infected with HIV and 90% of them were in Sub-Saharan Africa [1]. Children who are infected with HIV are known to be at high risk for poor nutritional status, infections and poor growth performance [2,3,4]. Growth failure in HIV-infected children is associated with an increased risk of mortality [5,6]. Episodes of diarrhea and of respiratory symptoms appear to be more common in HIV-infected children than in uninfected children [4].

Deficiencies of micronutrients, especially of vitamin A and zinc have been associated with diarrhea and respiratory diseases [7,8]. Micronutrient status also influences growth. Deficiencies of zinc and of iron have been associated with impaired growth [9,10]. Vitamin A and carotenoid status were demonstrated to be independent predictors of growth failure in HIV-infected Malawian children [11]. Micronutrient deficiencies seem to be common and more severe in HIV-infected children compared with uninfected ones [2,12].

Studies on the effect of micronutrient supplementation on growth and morbidity in malnourished children have not always resulted in undeniably positive outcomes. Vitamin A supplementation has been shown to be beneficial in reducing the risk of diarrhea, but may be detrimental against respiratory infections [7,13]. Zinc supplementation may improve growth [9] and reduce the incidence of diarrhea and respiratory infections in young children [14]. However, another study indicated that zinc supplementation increases respiratory infections [15], although adding vitamin A seems to reduce the adverse effects of zinc on respiratory infections [16]. Micronutrient deficiencies are seldom limited to a single micronutrient, therefore it has been suggested that supplements that contain an array of micronutrients should be provided [17].

Since HIV-infected children might be severely malnourished, they may profit most from the beneficial effects of multi-micronutrient administration. Unfortunately, such studies are scarce. The aim of the present study was to test the hypothesis that daily multi-micronutrient supplementation for six months would improve the weight-for-height and height-for-age *Z*-scores by 30% compared to placebo in HIV-infected young South African children who are not yet on antiretroviral therapy (ART). It was also hypothesized that the six months supplementation would reduce the number of episodes of diarrhea or respiratory symptoms by 30% in the same group of children.

## 2. Methods

The study was conducted from the Doctor George Mukhari hospital in Ga-Rankuwa, near Pretoria, the capital city of the Republic of South Africa.

HIV-infected children aged 4–24 months who had been previously admitted to the pediatric wards of the hospital with pneumonia or diarrhea were enrolled into the study, on discharge from the wards or from the pediatric outpatient department from November 2005 to November 2006.

Children who were on ART, or had received micronutrient supplementation (in the previous two months) or children with a chronic illness were not eligible for enrolment. At the time when the study was conducted, micronutrient supplementation was not standard care in HIV-infected children at the hospital. There were also concerns about zinc supplementation to HIV-infected children at the time.

The Medunsa Research Ethics and Publications Committee approved the study; the permission of the hospital authorities was obtained, and the parents or guardians provided signed informed consent. The children were referred to the ART clinic when necessary as per standard hospital procedure (immunological and clinical staging was used) and taken off the study when ARV therapy commenced. At the time of conducting the study, only children with CD4 percentage <15% or those with clinical stage 3 or 4 were eligible to receive ART.

In a double-blind, placebo controlled study; children were assigned to one of two intervention groups by computer randomization (1:1). The manufacturer prepared packs of tablets corresponding to the subject’s number according to the randomization schedule. The investigators, field workers and participants were blinded to the treatment assignments, which were revealed after completing data analysis. The children had monthly appointments at the hospital for follow up weight and height measurements, collection of tablets, and evaluation of side-effects of micronutrients.

At enrolment and at the third and sixth monthly hospital visits, blood samples were taken for serum zinc, retinol, iron and ferritin and CD4 T-lymphocyte counts.

The tablets were prepared by the pharmaceutical company Adminicle Trading (Edenvale, South Africa). The multi-micronutrient supplement was a crushable tablet which contained 300 µg retinol, 0.6 mg thiamin, 0.6 mg riboflavin, 8 mg niacin, 0.6 mg pyridoxine, 1 µg cobalamin, 70 µg folic acid, 25 mg ascorbic acid, 5 µg 1,25-dihydrocholecalciferol, 7 mg d,l-tocopherol, 700 µg copper, 8 mg iron, 30 µg selenium, and 8 mg zinc at amounts approximately based on recommended dietary allowances for a one year old child [18]. The placebo and supplement tablets were identical in appearance and taste. The caregiver crushed the tablet using a pill crusher and mixed it with a small portion of the breakfast cereal which was fed to the child before the rest of the meal.

### 2.1. HIV Tests

In children older than 15 months HIV-1 and HIV-2 serostatus was ascertained with ELISA tests [19]; in younger children a PCR was performed additionally. The CD4 T-lymphocyte counts were measured by means of a Coulter flow cytometer (Coulter Epics XL-MCL, Beckman Coulter, Johannesburg, South Africa) to stage the HIV infection.

### 2.2. Anthropometry

The subject’s age was calculated from the date of birth. The weight was measured with the child wearing only light clothing to the nearest 0.1 kg, using one digital scale, which was calibrated using a standard weight of 10 kg. The length was measured on a baby board in the recumbent position to 0.1 cm. *Z* scores for weight-for-age (WAZ), height-for-age (HAZ), and weight-for-height (WHZ) were calculated based on the World Health Organization standard values by means of the freely available Epi-Info Version 3.5.4 software.

### 2.3. Blood Sampling and Analysis

Blood samples (8 mL) were collected after an overnight fast by venipuncture (site cleaned with trace element free alcohol) from the cubital fossa or external jugular vein. Samples were collected in eight separate trace element free tubes, with a removable non-rubber lid protected from light, sent to the laboratory immediately after collection, and stored at −20 °C after centrifugation, until analysis. They were evaluated for hemolysis.

Serum zinc samples were measured by atomic absorption spectrometry (Perkin Elmer ICP/5500, Perkin Elmer Life and Analytical Sciences Inc., Waltham, MA, USA), serum retinol measurements by the fluorometric method [20], serum iron levels were measured by using rate spectrophotometry (SYNCHRON CX Systems IRON/TIBC Calibrator Kit, Beckman Instruments, Johannesburg, South Africa) and serum ferritin levels using commercial enzyme-linked immunoassay kits (Access Ferritin assay, Access Immunoassay Systems, Beckman Coulter, Johannesburg, South Africa). Quality control was performed by repeat analysis of standard reference material for low, normal and high values. The intra-assay and inter-assay coefficients of variation for serum zinc, retinol and ferritin were all less than 5%.

### 2.4. Morbidity

The children were visited at home twice a week by a field worker who assessed whether the child was suffering from diarrhea or symptoms of acute respiratory tract infection, and when the symptoms resolved. Compliance was evaluated by pill count. The children were referred to hospital when necessary. Diarrhea was present on any day when the child passed three or more loose stools in that day [21]. Acute respiratory symptoms were defined as the history of cough and fever.

### 2.5. Sample Size

A sample size calculation of 72 per group was calculated to be sufficient to detect a 30% difference in respiratory symptoms between placebo and supplement. The calculations were made considering a 5% level of significance and 80% power and based on information (standard deviation) from a study conducted on young Peruvian children [22]. In relation to improvement in weight-for-height and height-for-age *Z*-scores, the required sample sizes were 64 and 71, respectively. This was also based on the hypothesis that the supplement would result in a 30% improvement in these anthropometric indices when compared with placebo, at 5% significance and 80% power. The information was based on a study comparing the nutritional status of HIV-infected and HIV-uninfected children, which was conducted at the same hospital [2]. It was thus decided to enrol at least 90 per group to account for a possible 25% dropout rate; enrolling a total of 200 subjects was thus thought to be adequate.

## 3. Statistical Analysis

Data entry and analyses were performed on SPSS Statistics for Windows, version 18.0 [23]. Statistical analyses were two-tailed where appropriate and statistical significance was set at 5%. Data were assessed for normality and normalized by log transformation for serum ferritin.

The differences in anthropometric indices, and male to female ratios between the children who completed the study and those who dropped out were assessed by analysis of variance (ANOVA) and chi-square, respectively.

Anthropometric indices, micronutrient concentrations and immunological parameters were studied by means of ANOVA for repeated measures with treatment group and gender as between subjects’ factors. Changes in these measurements over 3 months and 6 months and differences between the two treatment groups were assessed using ANOVA by treatment group with age as a co-variant. The number of diarrheal and pneumonia episodes per month in the two treatment groups were compared using ANOVA by treatment group, age and gender.

## 4. Results

**Figure 1:**
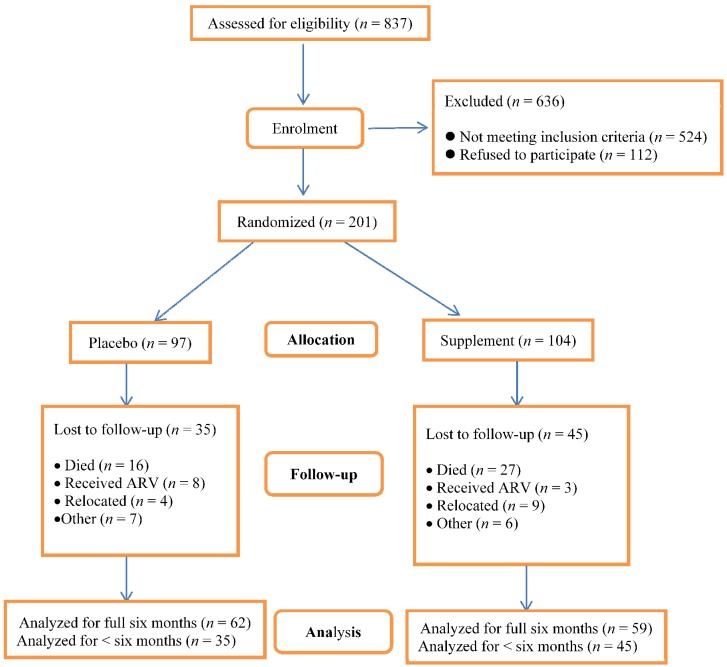
Assessment for eligibility, enrolment and losses to follow-up by treatment group. Analysis for the number of episodes of respiratory symptoms and of diarrhea was only conducted among children who were followed up for three or more months. The number of episodes was then divided by the number of months of follow-up. Analysis for changes in anthropometric indices, serum micronutrient concentrations and CD4 lymphocyte counts was only performed in children who had completed the study.

The number of children who were assessed for eligibility was 837; 636 were excluded and among these, 524 were not eligible. Out of the 524 who were not eligible, 486 of them were not HIV-infected, 17 were not in the eligible age group and 21 were on ART (see Figure 1). A total of 201 children aged 4–24 months were enrolled; 121 completed the study. Among the 80 children who dropped out (40%), 43 died, 11 received treatment with ART, 13 relocated to another area and another 13 dropped out for other reasons. Among the children who died, 80% died within the first month of follow up. The number of children who died and those who relocated was higher in the supplement group. The difference in mortality between the supplement and placebo group (27 *versus* 16, respectively) was not statistically significant (*p* = 0.12). On the other hand, the number of children who received ART was higher in the placebo group; this too was not statistically significant (*p* = 0.13).

The anthropometric indices and CD4 T-lymphocytes (at enrolment) of the study children who received the supplements and those who were given a placebo are compared in Table 1. The children who were given the supplements were 1.5 months older than those on placebos. In relation to the children who did not complete the six months follow up; there were no differences in the ages and the ratio of males to females compared with those who were followed up for the full six month period. The children who dropped out were significantly more stunted and underweight and marginally more wasted than those who completed the study, in both treatment groups. The percentages of CD4 T-lymphocytes of the children who dropped out were lower than those who completed the study; this however, was not statistically significant. Pill packets were returned at 294 of 372 scheduled visits (79%) for the placebo group by those who completed the study. Among those in the supplement group (who completed the study) pills were returned at 286 of 354 scheduled visits (81%); the difference was not statistically significant. The proportion of children with 2–5 pills was 8% and 9% in the placebo and supplement groups, respectively. There were no children with more than 5 pills in the pill packet.

**Table 1:** Anthropometric indices and CD4 T-lymphocytes of study children at enrolment.

Indices	Placebo	Supplement	All Study Children
*n*	97	104	201
Male/Female	43/54	51/53	94/107
Age (months)	12.02 ± 6.49	13.45 ± 6.66	12.76 ± 6.60
Weight (kg)	7.10 ± 1.83	7.20 ± 1.99	7.15 ± 1.92
Height (cm)	68.87 ± 7.86	70.61 ± 7.43	69.77 ± 7.67
WAZ	−2.12 ± 1.61	−2.42 ± 1.46	−2.28 ± 1.54
HAZ	−1.82 ± 1.79	−1.75 ± 1.66	−1.78 ± 1.72
WHZ	−1.23 ± 1.29	−1.71 ± 1.31	−1.48 ± 1.32
CD4 percent (*n*)	24.71 ± 12.67 (63)	23.89 ± 13.15 (57)	24.32 ± 12.85 (120)

Values presented as mean ± SD.

The immunological status of the children who completed the six months follow up was poor as denoted by the CD4 T-lymphocyte percentage (Table 2). At enrolment about 50% of the children in the placebo and supplement groups had CD4 T-lymphocyte proportions below 25%. The CD4 T-lymphocyte percentage did not improve within both treatment groups and there was no significant difference between the two groups.

**Table 2:** Immunological status of children during intervention period ^#^.

	Placebo		Supplement	
Follow Up	*n*	CD4 Percentage	*n*	CD4 Percentage
**Enrolment**	63	24.7 (21.6 to 27.9)	57	23.9 (20.5 to 27.3)
**3 mo**	48	27.1 (23.3 to 30.9)	41	23.9 (19.8 to 27.9)
**6 mo**	42	28.0 (24.2 to 31.7)	35	25.3 (20.8 to 29.9)
**Δ3mo**	48	−0.83 (−2.68 to 1.02)	41	−2.06 (−4.33 to 0.01)
**Δ 6 mo**	42	−1.44 (−3.71 to 0.83)	35	−0.64 (−3.39 to 2.11)

Values presented as mean (95% CI); ^#^ There were no significant differences between the two treatment groups in both absolute values and changes over 3 and 6 months.

The concentrations of serum zinc, retinol and ferritin over the 6 month period are presented in Table 3. There were no significant differences in the zinc and retinol concentrations between the two treatment groups at enrolment. The zinc concentrations improved significantly (by 14%) over the 6 month period within the supplement group, but not significantly within the placebo group. Nonetheless, there was no significant difference in the absolute change of serum zinc concentrations between the two treatment groups. The retinol concentrations did not differ significantly through the 6 month period within each treatment group and between the two treatment groups. The log ferritin increased by 0.1 in the supplement group. On the other hand, among children on placebos the log ferritin value deteriorated significantly.

**Figure 2:**
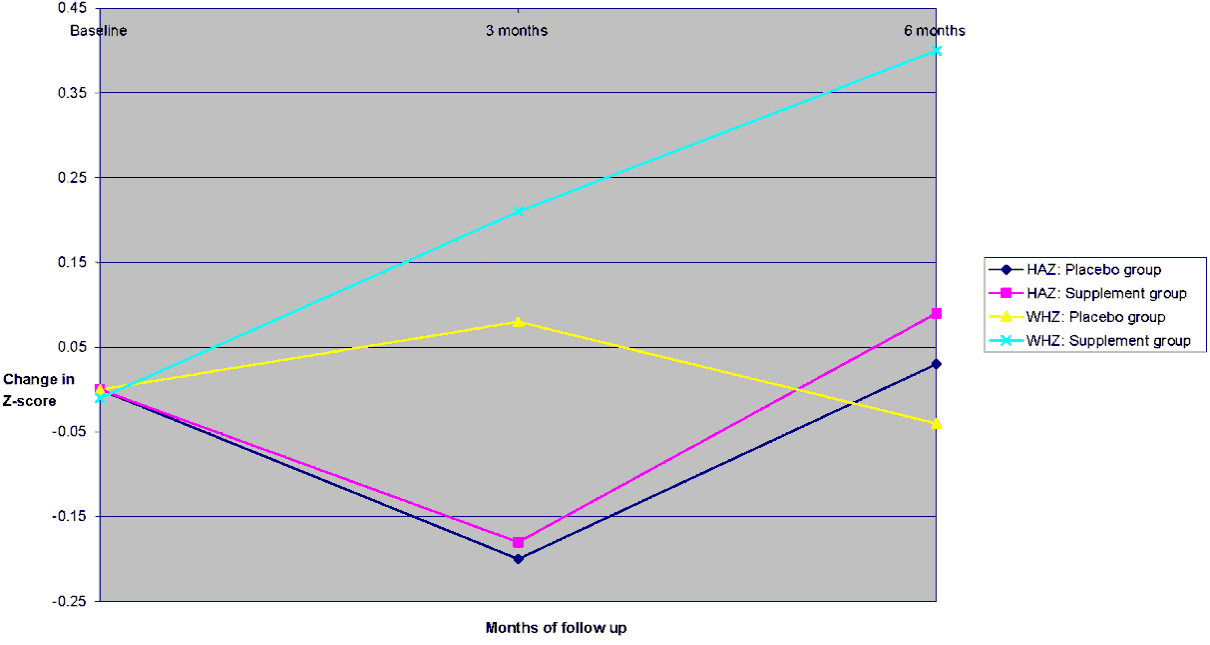
Change in anthropometrics *Z*-scores over the six month follow-up period among children who completed the study.

The anthropometric indices derived from the weights and heights (WAZ, HAZ and WHZ) of the children who completed the six month follow up period are shown in Table 4. The weights of the children increased significantly, by 1.35 kg in the placebo group and by 1.66 kg in the supplement group. Through the period of follow-up, there was a significant increase in height of 5.95 cm in the placebo group and 5.98 cm in the supplement group. Neither weight gain nor height increase was different between the two groups. There were no significant differences at enrolment between the two treatment groups in WAZ, HAZ and WHZ (Table 4), but the absolute low values of the *Z*-scores indicate that a large proportion of the children should be classified as wasted and stunted. WHZ as indicator for wasting and HAZ as indicator for stunting are graphically presented in Figure 2. Over the 6 month intervention there was a significant improvement in WAZ and WHZ values among the children in the supplement group but not in the placebo group. This improvement in WAZ and WHZ was shown to be independent of the children’s ages on ANOVA. The change in HAZ did not differ between the groups.

**Table 3:** Micronutrient status of the children during intervention period.

	Supplement			Placebo		
Follow Up	Zinc (µmol/L)	Retinol (µmol/L)	Log Ferritin (log[µg/L])	Zinc (µmol/L)	Retinol (µmol/L)	Log Ferritin (log[µg/L])
**Enrolment**	9.6 (8.8 to 10.5) (*n* = 75)	0.81 (0.70 to 0.95) (*n* = 62)	1.70 (1.58 to 1.81) (*n* = 75)	9.0 (8.3 to 9.7) (*n* = 78)	0.87 (0.75 to 0.99) (*n* = 53)	1.62 (1.52 to 1.73) (*n* = 78)
**3 mo**	9.8 (8.8 to 10.8) (*n* = 58)	0.78 (0.70 to 0.88) (*n* = 42)	1.54 (1.43 to 1.66) (*n* = 59)	10.4 (9.7 to 11.2) (*n* = 56)	0.84 (0.73 to 0.96) (*n* = 36)	1.66 (1.57 to 1.75) (*n* = 55)
**6 mo**	10.4 (9.5 to 11.3) (*n* = 53)	0.72 (0.65 to 0.79) (*n* = 34)	1.54 (1.40 to 1.68) (*n* = 52)	10.5 (9.6 to 11.4) (*n* = 51)	0.85 (0.70 to 1.0) (*n* = 32)	1.61 (1.51 to 1.71) (*n* = 49)
**Δ3mo**	−0.27 (−1.28 to 0.80) (*n* = 58)	−0.03 (−0.17 to 0.01) (*n* = 42)	−0.11 (−0.21 to −0.01) (*n* = 59)	1.17 (0.16 to 2.10) (*n* = 56)	0.01 (−0.16 to 0.15) (*n* = 36)	0.12 (0.03 to 0.21) (*n* = 55)
**Δ 6 mo**	0.41 (−0.67 to 1.50) (*n* = 53)	−0.05 (−0.21 to 0.11) (*n* = 34)	−0.06 (−0.17 to 0.05) (*n* = 52)	1.26 (0.14 to 2.39) (*n* = 51)	−0.01 (−0.24 to 0.22) (*n* = 32)	0.10 (−0.01 to 0.21) (*n* = 49)

Values presented as mean (95% CI).

**Table 4:** Anthropometric indices of the children during intervention period.

		Placebo						Supplement				
Follow Up		*n*	Age (mo)	WAZ	HAZ	WHZ		*n*	Age (mo)	WAZ	HAZ	WHZ
**Enrolment**		97	12.0 (10.7 to 13.3)	−2.12 (−2.44 to −1.80)	−1.82 (−2.18 to −1.46)	−1.23 (−1.49 to −0.97)		104	13.5 (12.2 to 14.7)	−2.42 (−2.70 to −2.14)	−1.75 (−2.07 to −1.43)	−1.71 (−1.96 to −1.46)
**1 mo**		77	13.5 (12.0 to 15.1)	−1.96 (−2.32 to −1.61)	−1.69 (−2.08 to −1.30)	−1.11 (−1.39 to −0.83)		73	15.0 (13.5 to 16.5)	−2.08 (−2.43 to − 1.73)	−1.50 (−1.83 to −1.17)	−1.48 (−1.77 to −1.19)
**2 mo**		72	14.5 (12.9 to 16.1)	−2.03 (−2.41 to −1.65)	−1.69 (−2.09 to −1.29)	−1.21 (−1.50 to −0.92)		70	16.3 (14.7 to 17.8)	−2.01 (−2.38 to −1.64)	−1.53 (−1.88 to −1.18)	−1.36 (−1.66 to −1.07)
**3 mo**		66	15.6 (14.2 to 17.0)	−1.90 (−2.30 to −1.50)	−1.63 (−2.04 to −1.22)	−1.08 (−1.37 to −0.79)		66	17.1 (15.5 to 18.7)	−1.96 (−2.34 to −1.58)	−1.51 (−1.87 to −1.15)	−1.31 (−1.64 to −0.98)
**4 mo**		62	16.6 (14.8 to 18.4)	−1.80 (−2.20 to −1.40)	−1.46 (−1.86 to −1.06)	−1.10 (−1.41 to −0.79)		62	18.1 (16.4 to 19.7)	−1.82 (−2.19 to −1.45)	−1.37 (−1.74 to 1.00)	−1.24 (−1.53 to −0.95)
**5 mo**		62	17.6 (15.8 to 19.4)	−1.84 (−2.25 to −1.43)	−1.45 (−1.88 to −1.02)	−1.17 (−1.48 to −0.86)		62	19.0 (17.3 to 20.6)	−1.78 (−2.15 to −1.41)	−1.35 (−1.73 to −0.97)	−1.20 (−1.50 to −0.90)
**6 mo**		62	18.5 (16.7 to 20.3)	−1.85 (−2.27 to −1.43)	−1.26 (−1.67 to −0.85)	−1.27 (−1.56 to −0.98)		59	20.0 (18.3 to 21.7)	−1.58 (−1.97 to –1.19)	−1.14 (−1.55 to −0.73)	−1.08 (−1.40 to −0.76)
**Δ3 mo**		66	3.5	−0.11 (−0.28 to 0.06)	−0.20 (−0.34 to −0.06) ^$^	0.08 (−0.15 to 0.31)		66	3.2	0.08 (−0.12 to 0.28) ^#^	−0.16 (−0.36 to 0.04)	0.22 (0.01 to 0.44) ^$^
**Δ 6 mo**		62	5.6	−0.19 (−0.46 to 0.08)	−0.03 (−0.25 to 0.19)	−0.04 (−0.39 to 0.31)		59	5.8	0.39 (0.12 to 0.66) ^$^^,#^	0.09 (−0.15 to 0.33)	0.40 (0.09 to 0.71) ^$^^,#^

Values presented as mean (95% CI) ^#^ Significantly different from placebo group (*p* < 0.05); ^$^ Significantly different from zero (*p* < 0.05).

The episodes of respiratory symptoms and of diarrhea were analyzed for children who had been followed up for 3 or more months. The number of episodes was then divided by the number of months of follow-up; these are presented in Table 5. The ages of these children were similar in the two treatment groups. The children who received the supplements had fewer episodes of diarrhea per month (0.25 (0.17–0.33)) (mean 95% CI) compared to those who were given placebos (0.36 (0.26–0.46)) (*p* = 0.09) and substantially lower number of episodes of respiratory symptoms: 0.66 (0.52–0.80) *versus* 1.01 (0.83–1.19) (*p* < 0.05); the latter difference was independent of the children’s ages on ANOVA.

**Table 5:** Diarrhea and respiratory symptom episodes per month of follow up.

Treatment Group	*n*	Age at Enrolment (months)	Number of Diarrheal Episodes per Month	Number of Episodes of Respiratory Symptoms per Month
Placebo	53	12.0 (10.3–13.7)	0.36 (0.26–0.46)	1.01 (0.83–1.19)
Supplement	52	13.7 (12.1–15.3)	0.25 (0.17–0.33) *	0.66 (0.52–0.80) ^#^

Values presented as mean (95% CI) * Marginally significantly different from placebo group (*p* = 0.09); ^#^ Significantly different from placebo group (*p* < 0.05).

## 5. Discussion

The objective of the study was to assess the impact of six month multi-micronutrient supplementation on growth performance and number of episodes of diarrhea and respiratory symptoms of HIV-infected children.

There was a high proportion of study children who did not complete the six month follow up period (40%); this can be considered as a limitation in this study. Among these children who did not complete the follow up period, the mortality was high, at 54% (21% of all enrolled children). The number of children who died was higher in the group that received the supplements (not statistically significant), and 80% of these children died within the first month of follow up in both treatment groups. However, the fact that most of the deaths occurred within the first month does not exclude the possibility that the micronutrients might have had a role in this. It is should also be noted that the children who received the supplements were also more wasted than those who were on the placebo; this finding was also not statistically significant. A pooled analysis of studies conducted in West, East and South Africa estimated that by two years of age, 53% of HIV-infected children would have died [24]. A survey that reviewed the mortality in a number of South African hospitals noted that approximately 48% of the deaths of children admitted to these hospitals were related to HIV infection [25]. Only 11 (14% of the total number of dropouts) children were commenced on ART in the current study. The ART program was only rolled out in South Africa in 2004. The relatively low proportion of children who received ART in the study may be related to the fact that the rollout of the ART program was still at an early stage.

Children who dropped out were significantly more stunted and more wasted at enrolment than those who completed the study in both groups but the difference in wasting approached significance only among children who received the supplement. This chance finding occurred in spite of careful randomization procedures. Poor growth has been linked with increased mortality in HIV-infected children. This is supported by a study that indicated that wasting and stunting in HIV-infected children are associated with increased mortality [5]. The immunological status (as measured by the percentage of CD4 T-lymphocytes) of the children who dropped out in the current study was significantly worse than that of those who completed the study. This finding is in line with a meta-analysis of 10 studies that suggested that CD4 measurements are the most important indicator of mortality in HIV-infected children not receiving ART [26].

Supplementation with micronutrients over a period of six months did not have an effect on the immunological status as measured by the CD4 T-lymphocytes in the current study. This is in keeping with a study in which zinc supplementation in HIV-infected young South African children had no effect on the percentage of CD4 lymphocytes when compared with a placebo [27]. In pregnant HIV-infected Tanzanian women, however, multivitamin supplementation significantly improved the women’s CD4 counts, compared with women who received a placebo [28].

The concentrations of serum zinc improved significantly over the study period among children who received the multi-micronutrient supplement. However, the absolute change in serum zinc and retinol concentrations was not significantly different between the two treatment groups. On the other hand was a significant difference in the change in log ferritin (it was higher in the supplement group). Unfortunately, blood samples for acute phase reactants (e.g., C-reactive protein) were not taken; this might have hampered the assessment of the difference in micronutrient concentrations.

In the current study, the improvement in WAZ and WHZ over the six month period was significantly greater among children who were given the supplements. However, the supplements had no effect on stunting. It is important to note that the children who received the supplements were 1.5 months older than those who were on the placebo. Nonetheless, the improvement in WAZ and WHZ was independent of age. In Peru (an area with low HIV prevalence), no improvement was noted in WAZ and HAZ in children who were given multi-micronutrients [22]. Supplementation with multiple micronutrients combined with vitamin A in stunted South African children (6–24 months of age) significantly improved height, compared with vitamin A alone or vitamin A plus zinc [29]. It has been suggested that micronutrient supplements are more effective in children who have poor anthropometric indices. The children in the current study were more wasted and stunted than the children in the above noted studies. The multi-micronutrients were given for six months in both the current and the Peruvian studies. However, in the study with stunted South African children, the supplements were given for 18 months. It is thus conceivable that the six month period of supplementation in the current study might have been too short.

In the current study, children who received the micronutrient supplement had significantly fewer episodes of respiratory symptoms and fewer episodes of diarrhea than those who were on the placebo; the difference in diarrheal episodes did not reach statistical significance. In young South African children (6–24 months of age) who were born to HIV-infected mothers, receipt of a multi-micronutrient supplement (that included vitamin A) did not reduce the prevalence of diarrhea or respiratory symptoms when compared with children who were given vitamin A only irrespective of HIV status [30]. However, multi-micronutrient supplementation reduced the incidence of diarrhea in rural South African children compared with those who received vitamin A only; the reduction was only noted in stunted HIV-uninfected children [31]. Similarly, zinc supplements significantly reduced episodes of diarrhea in HIV-infected South African children; episodes of pneumonia, however, were marginally reduced [26]. A meta-analysis of 17 studies (from areas with low HIV prevalence) concluded that zinc supplementation significantly reduced the risk of diarrhea and respiratory illnesses [32].

## 6. Conclusions

The improvement in growth performance (in weight but not height) and reduction in morbidity that was observed in this study suggests that micronutrient supplements are useful as adjunct therapy in HIV-infected children. While there was no effect on CD4 lymphocytes, there were no deleterious effects. At the time of conducting the study, ART was not widely available at the local hospital, but has since become available to an increasing number of children in sub-Saharan African countries. However, because ART is given to children with advanced HIV disease, micronutrient supplementation may be useful in those children who are as yet not eligible for ART. The effect of micronutrient supplementation in children who are on ART should also be investigated.
